# The association between the polymorphism of RAR related orphan receptor beta and the clinical manifestations of oral squamous cell carcinoma

**DOI:** 10.7150/jca.103945

**Published:** 2025-01-01

**Authors:** Wei-En Yang, Chiao-Wen Lin, Yi-Tzu Chen, Chun-Wen Su, Chia-Ming Yeh, Chia-Yi Lee, Shun-Fa Yang, Mu-Kuan Chen

**Affiliations:** 1 Institute of Medicine, Chung Shan Medical University, Taichung, Taiwan.; 2 Department of Medical Research, Chung Shan Medical University Hospital, Taichung, Taiwan.; 3 Institute of Oral Sciences, Chung Shan Medical University, Taichung, Taiwan.; 4 Department of Dentistry, Chung Shan Medical University Hospital, Taichung, Taiwan.; 5 Nobel Eye Institute, Taipei, Taiwan.; 6 Department of Otorhinolaryngology, Head and Neck Surgery, Changhua Christian Hospital, Changhua, Taiwan.; 7 Department of Post-Baccalaureate Medicine, College of Medicine, National Chung Hsing University, Taichung, Taiwan.

**Keywords:** single nucleotide polymorphism, RAR related orphan receptor beta, oral squamous cell carcinoma, betel quid chewing, buccal mucosa cancer, oral cancer

## Abstract

Oral squamous cell carcinoma (OSCC) affects a substantial proportion of the Asian population and is influenced by various genetic risk factors. The *RAR-related orphan receptor beta* (*RORB*), a regulator of the circadian rhythm, has been implicated in certain neoplasms. Accordingly, this study investigated the association between *RORB* single-nucleotide polymorphisms and clinical manifestations of OSCC. A total of 1174 male patients without OSCC and 1254 male patients with OSCC were included in the study. Three *RORB* single-nucleotide polymorphism loci—rs3750420 (T/C), rs10781247 (A/G), and rs17611535 (C/T)—were genotyped using TaqMan allelic discrimination assays. *RORB* single-nucleotide polymorphism rs10781247 variants were significantly associated with moderate to poor cellular differentiation in patients with OSCC (*p* = 0.042). Additionally, among betel quid chewers with OSCC, rs10781247 variants were significantly associated with moderate to poor cell differentiation (*p* = 0.036). The rs3750420 variants were significantly associated with larger tumor size in individuals with buccal mucosa cancer (*p* = 0.036). An analysis of Cancer Genome Atlas data revealed that *RORB* mRNA levels were significantly higher in patients with head and neck squamous cell carcinoma compared with controls (*p* = 0.0002). Moreover, *RORB* mRNA levels were significantly higher in stage IV tumors than in stage III tumors (*p* = 0.0252). In conclusion, *RORB* single-nucleotide polymorphisms rs3750420 and rs10781247 are associated with adverse clinical characteristics in OSCC.

## Introduction

Oral squamous cell carcinoma (OSCC) is a common malignancy worldwide that is characterized by epithelial dysplasia 1, 2. In Asia, the prevalence of OSCC among men is approximately 21.2 per 100,000 3. The most common subtypes of OSCC include buccal cancer, mouth floor cancer, and tongue cancer. Hard palate cancer represents a relatively minor subset of OSCC 4, 5. Current treatment modalities for OSCC are surgical excision, chemotherapy, radiotherapy, and targeted therapy 6. The 5-year survival rate for OSCC is approximately 60%, but this declines to approximately 40% in stage IV cases 3, 7.

Several risk factors for OSCC have been identified 8, 9. Cigarette smoking and betel quid chewing are two prominent lifestyle-related risk factors for OSCC, particularly in the Asian population 10. Human papillomavirus is another risk factor, accounting for the majority of OSCC cases 11. Regarding biomarkers, PD-L1 and interleukins have been implicated in OSCC development and can serve as potential indicators of the disease 12. From a genetic perspective, mutations in the *p53* gene are strongly associated OSCC pathogenesis 13. However, other potential risk factors for OSCC remain to be elucidated and warrant further investigation.

*RAR-related orphan receptor beta* (*RORB*) is a protein-coding gene that regulates circadian rhythms in the human body 14. *RORB* was previously demonstrated to be correlated with bipolar disorder 15. Moreover, *RORB* expression was significantly correlated with alterations in the tumor immune microenvironment in head and neck squamous cell carcinoma 16. In the context of neoplasms, *RORB* polymorphisms were associated with the development of breast cancer 17. Evidence of the relationship between *RORB* polymorphisms and OSCC is limited. Because *RAR-related orphan receptor alpha* (*RORA*) has been implicated in the pathophysiology of OSCC 18, *RORB*, as part of the same receptor family, along with its single-nucleotide polymorphisms (SNPs), may also play a role in OSCC pathogenesis.

The present study evaluated the association between *RORB* SNPs and OSCC occurrence. Additionally, the relationship between *RORB* polymorphisms and OSCC across different populations was analyzed.

## Materials and Methods

### Ethics declarations

All procedures conducted in the present study adhered to the principles outlined in the Declaration of Helsinki (1964) and its subsequent amendments. The study protocol was reviewed and approved by the Institutional Review Board of Chung Shan Medical University Hospital (project identification code: CS1-21151). Written informed consent was obtained from all participants after a thorough explanation of the study objectives and procedures.

### Medical data review and sample collection

The study included 1174 and 1254 male patients without and with OSCC, respectively. Basic demographic and clinical information, including age, OSCC stage, TNM stage, and degree of cell differentiation, were extracted from medical records held at Chung Shan Medical University Hospital. Only the most recent OSCC stage and TNM stage of each patient were used in the analysis. Regarding laboratory sample collection, venous blood samples were obtained from all participants. The blood samples were collected in ethylenediaminetetraacetic acid tubes, centrifuged, and stored in an experimental refrigerator at approximately -80°C, following protocols described elsewhere 19.

### DNA analysis of RORB SNPs by using real-time PCR

The *RORB* SNPs examined in this study were rs3750420 (T/C), rs10781247 (A/G), and rs17611535 (C/T). These three *RORB* SNPs were selected because their minor allele frequencies exceeded 5% and because they were demonstrated to be associated with breast cancer and other disorders 14, 15, 20. DNA extraction and analysis methods followed protocols established in other studies by the same authors 21. Genomic DNA was isolated from leukocyte samples by using QIAamp DNA kits (Qiagen, Valencia, Valencia, CA, USA) following the manufacturer's instructions. Isolated DNA was stored at approximately -20°C until analysis. Genotyping of the three *RORB* SNPs was performed using the ABI StepOne Real-Time PCR System (Applied Biosystems, Foster City, CA, USA). The results were analyzed using SDS software version 3.0 (Applied Biosystems).

### Bioinformatics analysis of RORB mRNA levels

To explore the association between *RORB* mRNA levels and clinical characteristics of OSCC, we used data from the Cancer Genome Atlas, a global genomic database 22. In this analysis, patients with head and neck squamous cell carcinoma were stratified into subgroups on the basis of tumor presence and tumor stage.

### Statistical analysis

Statistical analyses were conducted using SAS software version 9.4 (SAS Institute, NC, USA). Descriptive analyses were performed to summarize the demographic and tumor-related data of the two study groups. The chi-square test was employed to compare each group's demographic characteristics. A multiple logistic regression model was employed to examine the distribution frequency of each *RORB* SNP in each group after controlling for age, betel quid chewing, cigarette smoking, and alcohol consumption. Subsequently, the clinical characteristics of patients with OSCC with different genotypes of *RORB* SNP rs10781247 were analyzed using multiple logistic regression. The patients with OSCC were further stratified into betel quid chewers and non-betel quid chewers, and the association between *RORB* SNP genotypes and clinical characteristics of OSCC was reanalyzed using multiple logistic regression. A *p* value of <0.05 was considered significant.

## Results

### Baseline characteristics of each group

Baseline characteristics are presented in Table 1. The proportion of patients aged >60 years was significantly higher in the OSCC group than in the control group (*p* = 0.019). Additionally, the prevalence of betel quid chewing, cigarette smoking, and alcohol consumption was significantly greater in the OSCC group than in the control group (all *p* < 0.001). Overall, the OSCC group had a higher tumor stage and more advanced tumor T status than did the control group (Table 1).

### Genotype frequencies of RORB SNPs in each group

In the multiple logistic regression analysis, the distributions of all three *RORB* SNPs exhibited no significant differences between the OSCC and control groups (all *p* > 0.05; Table 2). However, the *RORB* SNP rs10781247 variants were significantly associated with moderate to poor cell differentiation in the OSCC group (AOR: 1.403, 95% CI: 1.011 to 1.947, *p* = 0.042; Table 3). Additionally, the *RORB* SNP rs10781247 variants were significantly associated with moderate to poor cell differentiation in the patients with OSCC who were betel quid chewers (AOR: 1.486, 95% CI: 1.025 to 2.155, *p* = 0.036), whereas no significant association was observed between *RORB* SNPs and clinical characteristics of patients with OSCC who were nonbetel quid chewers (all *p* > 0.05; Table 4). Moreover, *RORB* SNP rs3750420 variants were significantly associated with larger tumor size in individuals with buccal mucosa cancer (AOR: 1.542, 95% CI: 1.027 to 2.315, *p* = 0.036). However, no significant association was observed between *RORB* SNP rs3750420 genotypes and clinical characteristics of OSCC in the participants with tongue cancer (all *p* > 0.05; Table 5).

### Bioinformatics analysis of RORB expression in head and neck squamous cell carcinoma

**Figure 1:**
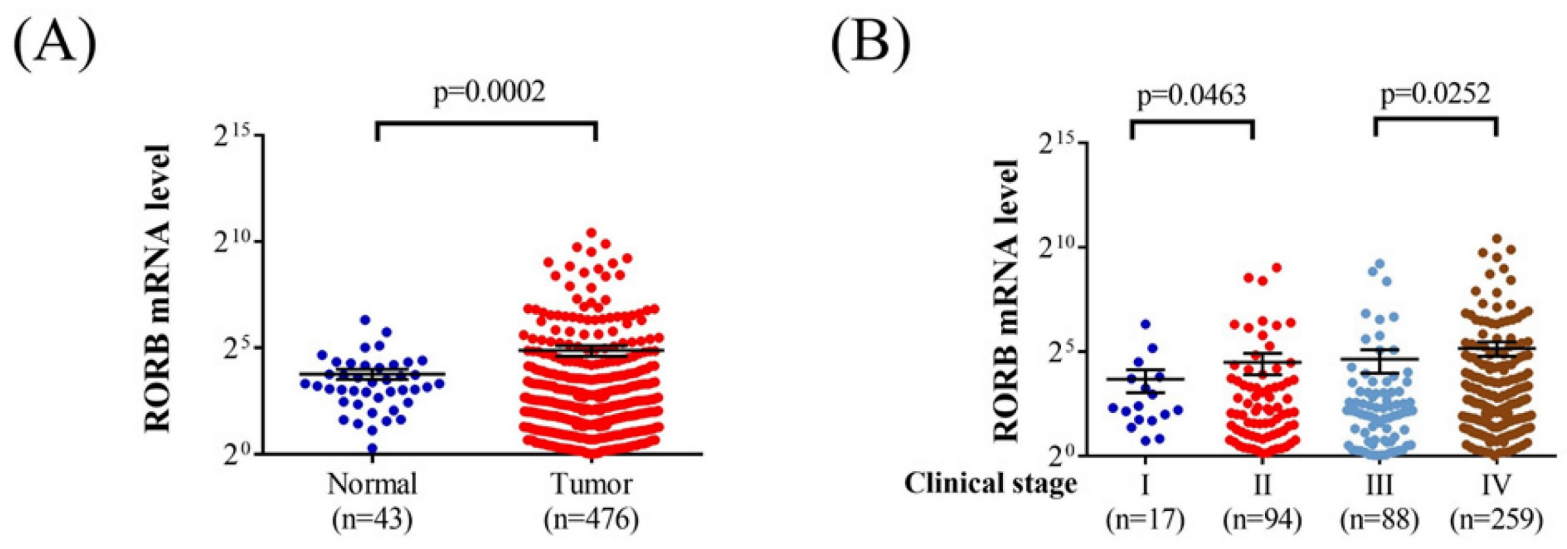
** The Analysis of RORB mRNA by The Cancer Genome Atlas database.** (A) The RORB mRNA level in head and neck squamous cell carcinoma patients and noncancerous tissues. (B) The RORB mRNA level in head and neck squamous cell carcinoma patients with different tumor stage.

Analysis of Cancer Genome Atlas data revealed that *RORB* mRNA levels were significantly higher in patients with head and neck squamous cell carcinoma than in those with noncancerous tissues (*p* = 0.0002; Figure 1A). Furthermore, *RORB* mRNA levels were significantly higher in the stage II tumors than in stage I tumors (*p* = 0.0463). Moreover, the *RORB* mRNA levels were significantly higher in stage IV tumors than in stage III tumors (*p* = 0.0252; Figure 1B).

## Discussion

In the present study, variants of the *RORB* SNP rs10781247 were associated with moderate to poor cell differentiation in both the overall OSCC group and the subgroup of patients with OSCC with a history of betel quid chewing. Moreover, variants of the *RORB* SNP rs3750420 were associated with a higher tumor T status in patients with buccal mucosa cancer. Furthermore, *RORB* mRNA levels were significantly higher in patients with head and neck squamous cell carcinoma, with the highest expression levels observed in those with advanced tumor stages.

The *RORB* gene has been implicated in various diseases 14, 15. The *RAR-related orphan receptor* (*ROR*) family regulates the circadian rhythm and influences sleep patterns, with sleep duration affected by *RORA* and its SNP rs75981965 23. Additionally, the *ROR* family plays a role in the aging process, where both *RORA* and *RORB* independently contribute to the risk of cognitive aging 14. In addition to circadian regulation, the *ROR* family is involved in the growth of specific cell types, such as epithelial cells and osteocytes 24, and research has demonstrated that *RORA* expression is suppressed during the development of OSCC 18. The expression of *RORB* is also correlated with prognosis in breast cancer 25, with lower levels observed in the patients with endometrial cancer 24. Zheng *et al.*, reported that *RORB* expression was significantly associated with changes in the tumor immune microenvironment in head and neck squamous cell carcinoma 16. Beyond gene expression, the genetic polymorphisms of *RORB* can influence disease development. For instance, the *RORB* SNP rs7867494 is associated with an increased incidence of breast cancer 17, and multiple *RORB* SNP variants are associated with the development of lung and prostate cancers 26. In addition to the *RORB* gene family, several other genetic factors contribute to the development of OSCC 13, 27, 28. The presence of *CDKN2A* was found to be significantly correlated with OSCC development 29, and mutations in the *p53* gene are commonly observed in patients with OSCC 27. Genetic polymorphisms, such as the *interleukin-10* SNP-A592C, are more frequently observed in individuals with OSCC than in wild-type carriers 30, and the SNP rs1412115 A>G on chromosome 10 was demonstrated to increase the risk of OSCC in a Chinese population 31. Additionally, *GAS5* SNP rs145204276 (Ins/Del or Del/Del) variants are associated with a higher risk of moderate to poor cell differentiation in oral cancer 32. Because both *RORB* and its SNPs have been demonstrated to regulate cancer growth 17, 24 and because *RORA* levels are significantly higher in individuals with OSCC 18, *RORB* SNP variants may be corrected with the clinical status of OSCC. This hypothesis is supported by the results of the present study.

In the present study, the distribution of *RORB* SNP rs10781247 variants was more commonly found in patients with OSCC with moderate to poor cell differentiation. Another study also reported that *RORB* SNP rs10781247 variants were more common in individuals with cognitive aging 14. The association between *RORB* SNP rs10781247 variants and OSCC has not been extensively explored. To the best of our knowledge, this study provides preliminary evidence linking the distribution frequency of *RORB* SNP rs10781247 variants with cell differentiation in OSCC. Moreover, this study solely focused on male patients because male sex is a known risk factor for OSCC 33. Several confounders, including age, betel quid chewing, cigarette smoking, and alcohol consumption, were adjusted in the multiple logistic regression model 3, 10. Consequently, *RORB* SNP rs10781247 variants were independently associated with poorer cell differentiation in patients with OSCC. The *RORB* gene has been shown to increase the proliferation of retinal cells and neurons 24, 34, suggesting that *RORB* SNP rs10781247 variants may exert a similar effect on OSCC cells. Additionally, the OSCC subgroup with a history of betel quid chewing demonstrated a strong association between *RORB* SNP rs10781247 variants and moderate to poor cell differentiation. Betel quid chewing is a well-established risk factor for OSCC 35, and research has demonstrated that it contributes to a higher incidence of OSCC, particularly in buccal mucosa cancer 3. Therefore, the synergic effect of betel quid chewing and *RORB* SNP rs10781247 variants may lead to worse differentiation of OSCC cells. By contrast, patients with OSCC with *RORB* SNP rs10781247 variants but no history of betel quid chewing did not exhibit an increased risk of poor cell differentiation during OSCC development.

In addition to the *RORB* SNP rs10781247 variants, a significantly higher distribution frequency of *RORB* SNP rs3750420 variants was observed in patients with buccal mucosa cancer and a higher tumor T status compared with individuals with tongue cancer. Although both buccal mucosa cancer and tongue cancer are types of OSCC, some differences exist between these two neoplasms 10. The treatment approaches for buccal mucosa cancer and tongue cancer are also slightly different, with concurrent chemoradiotherapy providing additional benefits for tongue cancer 3. Moreover, the 5-year distal metastasis rate for buccal mucosa cancer with nodal involvement is 30%, which is significantly higher than the 18% for tongue cancer with nodal invasion 36. Genetically, buccal mucosa cancer has been associated with polymorphisms in the *LINC00312* gene 37, whereas the stage of tongue cancer is correlated with the SNP rs9904341 of the *survivin* gene 38. We speculated that the *RORB* SNP rs3750420 variants interact with genes involved in the development of buccal mucosa cancer, thereby influencing the clinical characteristics of this cancer type. Additionally, a study demonstrated that *RORB* SNP rs3750420 variants are associated with the development of breast cancer 20, which could also explain the larger tumor size and tumor progression observed in buccal mucosa cancer in patients with this particular variant.

In the bioinformatics analysis using the Cancer Genome Atlas database, individuals with head and neck squamous cell carcinoma exhibited significantly higher *RORB* mRNA expression compared with noncancerous tissues. Moreover, *RORB* mRNA expression was highest in stage IV head and neck squamous cell carcinoma and lowest in stage I head and neck squamous cell carcinoma. A study demonstrated that lower *RORA* expression is associated with OSCC development 18. The bioinformatics analysis in the present study aligns with these findings, suggesting that the expression of the *ROR* gene family is correlated with OSCC development. Although OSCC does not fully represent head and neck squamous cell carcinoma 39, it constitutes a substantial proportion of cases within the broader head and neck squamous cell carcinoma population and ranks sixteenth in cancer incidence worldwide 10, 35, 40. Additionally, the higher expression of *RORB* mRNA in advanced OSCC stages indicates that the *RORB* gene may shape the clinical characteristics of OSCC. By integrating the results from the bioinformatics analysis and the clinical data from patients with OSCC in this study, we hypothesize that genetic polymorphisms of *RORB* SNPs are attributable to variations in *RORB* mRNA expression, leading to higher levels of *RORB* mRNA in specific OSCC subgroups. However, further research is warranted to fully elucidate this hypothesis.

This study has several limitations. First, the study employed a case-control design rather than a prospective cohort design, limiting our ability to evaluate the progression and prognosis of OSCC. Second, the analysis did not include data on the dose and duration of betel quid chewing, cigarette smoking, and alcohol consumption because some patients did not provide detailed information regarding these habits. This absence of data may have compromised the completeness and accuracy of the analyses and results. Additionally, the population in the Cancer Genome Atlas database significantly differs from our study cohort, which may introduce considerable heterogeneity in the results. Finally, because nearly all the participants in this study were Han Taiwanese, the external validity of the findings may be limited, reducing the generalizability to other populations.

In conclusion, the presence of *RORB* SNP rs10781247 variants and *RORB* SNP rs3750420 variants was associated with poorer clinical characteristics of OSCC in specific populations. Furthermore, the *RORB* mRNA expression levels were significantly higher in patients with head and neck squamous cell carcinoma, particularly in those with advanced tumor stages. Consequently, more frequent follow-up is recommended for patients with OSCC with *RORB* SNP rs10781247 and rs3750420 variants for monitoring potential adverse tumor progression. Further large-scale, prospective studies are essential to investigate the correlation between *RORB* SNPs and therapeutic outcomes in OSCC.

## Figures and Tables

**Table 1:** The distributions of demographical characteristics in 1174 controls and 1254 male patients with oral squamous cell carcinoma.

Variable	Control group (N=1174)	OSCC group (N=1254)	*p* value
Age (years)			
< 60	761 (64.8%)	755 (60.2%)	0.019*
≥ 60	413 (35.2%)	499 (39.8%)	
Betel quid chewing			
No	978 (83.3%)	398 (31.7%)	
Yes	196 (16.7%)	856 (68.3%)	< 0.001*
Cigarette smoking			
No	552 (47.0%)	260 (20.7%)	
Yes	622 (53.0%)	994 (79.3%)	< 0.001*
Alcohol drinking			
No	942 (80.2%)	773 (61.6%)	
Yes	232 (19.8%)	481 (38.4%)	< 0.001*
Stage			
I+II		566 (45.1%)	
III+IV		688 (54.9%)	
Tumor T status			
T1+T2		604 (48.2%)	
T3+T4		650 (51.8%)	
Lymph node status			
N0		850 (67.8%)	
N1+N2+N3		404 (32.2%)	
Metastasis			
M0		1247 (99.4%)	
M1		7 (0.6%)	
Cell differentiation			
Well differentiated		204 (16.3%)	
Moderately or poorly differentiated		1050 (83.7%)	

N: number, OSCC: oral squamous cell carcinoma Mann-Whitney U test was used between healthy controls and patients with oral cancer. * p value < 0.05 as statistically significant.

**Table 2:** Odds ratio and 95% confidence interval of oral squamous cell carcinoma associated with *RORB* genotypic frequencies.

Variable	Controls (N=1174) (%)	Patients (N=1254) (%)	AOR (95% CI)^#^	*p* value
**rs3750420**				
TT	380 (32.4%)	408 (32.5%)	1.000 (reference)	
TC	597 (50.9%)	617 (49.2%)	0.944 (0.765-1.166)	0.593
CC	197 (16.7%)	229 (18.3%)	0.995 (0.753-1.314)	0.970
TC+CC	794 (67.6%)	846 (67.5%)	0.957 (0.784-1.168)	0.665
**rs10781247**				
AA	312 (26.6%)	322 (25.7%)	1.000 (reference)	
AG	598 (50.9%)	650 (51.8%)	1.194 (0.953-1.497)	0.124
GG	264 (22.5%)	282 (22.5%)	1.148 (0.876-1.504)	0.317
AG+GG	862 (73.4%)	932 (74.3%)	1.180 (0.952-1.462)	0.130
**rs17611535**				
CC	1004 (85.5%)	1093 (87.2%)	1.000 (reference)	
CT	159 (13.5%)	156 (12.4%)	0.923 (0.699-1.219)	0.573
TT	11 (1.0%)	5 (0.4%)	0.361 (0.106-1.226)	0.102
CT+TT	170 (14.5%)	161 (12.8%)	0.884 (0.673-1.160)	0.372

AOR: adjusted odds ratio, CI: confidence intervals, N: number ^#^ The adjusted odds ratio with their 95% confidence intervals were estimated by multiple logistic regression models after controlling for age, betel quid chewing, cigarette smoking, and alcohol drinking.

**Table 3:** Odds ratio and 95% confidence intervals of clinical statuses associated with genotypic frequencies of *RORB* rs10781247 in male oral squamous cell carcinoma patients.

Variable	AA (N=322)	AG+GG (N=932)	AOR (95% CI)^#^	*p* value
**Clinical Stage**				
Stage I+II	154 (47.8%)	412 (44.2%)	1.000 (reference)	0.260
Stage III+IV	168 (52.2%)	520 (55.8%)	1.157 (0.897-1.492)	
**Tumor size**				
≦ T2	161 (50.0%)	443 (47.5%)	1.000 (reference)	0.445
> T2	161 (50.0%)	489 (52.5%)	1.104 (0.857-1.422)	
**Lymph node metastasis**				
No	221 (68.6%)	629 (67.5%)	1.000 (reference)	0.705
Yes	101 (31.4%)	303 (32.5%)	1.054 (0.803-1.384)	
**Metastasis**				
M0	321 (99.7%)	926 (99.4%)	1.000 (reference)	0.489
M1	1 (0.3%)	6 (0.6%)	2.080 (0.249-17.342)	
**Cell differentiated grade**				
Well	64 (19.9%)	140 (15.0%)	1.000 (reference)	0.042*
Moderate or poor	258 (80.1%)	792 (85.0%)	1.403 (1.011-1.947)	

AOR: adjusted odds ratio, CI: confidence intervals, N: number ^#^ The adjusted odds ratio with their 95% confidence intervals were estimated by logistic regression models. * *p* value < 0.05 as statistically significant.

**Table 4:** Clinical statuses and genotypic frequencies of *RORB* rs10781247 in oral squamous cell carcinoma who are betel quid chewers or not betel quid chewers.

Variable	Betel Quid Chewers (N=856)				Non-Betel Quid Chewers (N=398)			
	AA (N=230)	AG+GG (N=626)	AOR (95% CI)^#^	*p* value	AA (N=92)	AG+GG (N=306)	AOR (95% CI)^#^	*p* value
**Clinical Stage**								
Stage I+II	110 (47.8%)	289 (46.2%)	1.000 (reference)	0.666	44 (47.8%)	123 (40.2%)	1.000 (reference)	0.193
Stage III+IV	120 (52.2%)	337 (53.8%)	1.069 (0.790-1.447)		48 (52.2%)	183 (59.8%)	1.364 (0.854-2.179)	
**Tumor size**								
≤ T2	111 (48.3%)	310 (49.5%)	1.000 (reference)	0.744	50 (54.3%)	133 (43.5%)	1.000 (reference)	0.066
> T2	119 (51.7%)	316 (50.5%)	0.951 (0.703-1.287)		42 (45.7%)	173 (56.5%)	1.549 (0.969-2.474)	
**Lymph node metastasis**								
No	159 (69.1%)	426 (68.1%)	1.000 (reference)	0.763	62 (67.4%)	203 (66.3%)	1.000 (reference)	0.851
Yes	71 (30.9%)	200 (31.9%)	1.051 (0.759-1.457)		30 (32.6%)	103 (33.7%)	1.049 (0.638-1.722)	
**Metastasis**								
M0	229 (99.6%)	621 (99.2%)	1.000 (reference)	0.572	92 (100.0%)	59 (99.7%)	1.000 (reference)	---
M1	1 (0.4%)	5 (0.8%)	1.844 (0.214-15.866)		0 (0.0%)	1 (0.3%)	---	
**Cell differentiation**								
Well	53 (23.0%)	105 (16.8%)	1.000 (reference)	0.036*	11 (12.0%)	35 (11.4%)	1.000 (reference)	0.891
Moderate or poor	177 (77.0%)	521 (83.2%)	1.486 (1.025-2.155)		81 (88.0%)	271 (88.6%)	1.051 (0.511-2.164)	

AOR: adjusted odds ratio, CI: confidence intervals, N: number ^#^ The adjusted odds ratio with their 95% confidence intervals were estimated by multiple logistic regression models after controlling for cigarette smoking, and alcohol drinking. * *p* value < 0.05 as statistically significant.

**Table 5:** Clinical statuses and genotypic frequencies of *RORB* rs3750420 in buccal mucosa cancer and tongue cancer

Variable	Buccal mucosa cancer (N=433)				Tongue cancer (N=409)			
	TT (N=140)	TC+CC (N=293)	AOR (95% CI)^#^	*p* value	TT (N=130)	TC+CC (N=279)	AOR (95% CI)^#^	*p* value
**Clinical Stage**								
Stage I+II	69 (49.3%)	133 (45.4%)	1.000 (reference)	0.448	62 (47.7%)	126 (45.2%)	1.000 (reference)	0.632
Stage III+IV	71 (50.7%)	160 (54.6%)	1.169 (0.781-1.750)		68 (52.3%)	153 (54.8%)	1.107 (0.729-1.680)	
**Tumor size**								
≤ T2	81 (57.9%)	138 (51.8%)	1.000 (reference)	0.036*	66 (50.8%)	141 (50.5%)	1.000 (reference)	0.965
> T2	59 (42.1%)	155 (52.9%)	1.542 (1.027-2.315)		64 (49.2%)	138 (49.5%)	1.009 (0.666-1.530)	
**Lymph node metastasis**								
No	98 (70.0%)	205 (70.0%)	1.000 (reference)	0.994	79 (60.8%)	174 (62.4%)	1.000 (reference)	0.757
Yes	42 (30.0%)	88 (30.0%)	1.002 (0.645-1.554)		51 (39.2%)	105 (37.6%)	0.935 (0.610-1.433)	
**Metastasis**								
M0	139 (99.3%)	292 (99.7%)	1.000 (reference)	0.592	130 (100.0%)	277 (99.3%)	1.000 (reference)	---
M1	1 (0.7%)	1 (0.3%)	0.476 (0.030-7.667)		0 (0.0%)	2 (0.7%)	---	
**Cell differentiation**								
Well	22 (15.7%)	55 (18.8%)	1.000 (reference)	0.436	17 (13.1%)	33 (11.8%)	1.000 (reference)	0.720
Moderate or poor	118 (84.3%)	238 (81.2%)	0.807 (0.469-1.386)		113 (86.9%)	246 (88.2%)	1.121 (0.600-2.097)	

AOR: adjusted odds ratio, CI: confidence intervals, N: number ^#^ The adjusted odds ratio with their 95% confidence intervals were estimated by multiple logistic regression models after controlling for cigarette smoking, and alcohol drinking. * *p* value < 0.05 as statistically significant.
